# Concreteness Effects of Constituents in Naming Mandarin Compounds

**DOI:** 10.1007/s00426-026-02278-6

**Published:** 2026-03-26

**Authors:** Jiaqi Wang, Niels O. Schiller, Claartje Levelt

**Affiliations:** 1 The School of Foreign Languages, North China Electric Power University, Beijing, China; 2 Department of Linguistics and Translation, City University of Hong Kong, Kowloon, Hong Kong; 3 Leiden University Centre for Linguistics, Leiden University, Leiden, The Netherlands

## Abstract

The present study aims to use the concreteness effect to provide a detailed investigation of how Mandarin compounds are represented in the mental lexicon, that is, whether abstract and concrete morpheme constituents can influence the retrieval of Mandarin compounds during production. Our study investigated the question of the representation of Mandarin compound words through a picture naming task where forty-one participants were recruited. The behavioral outcomes indicated that there was a constituent concrete effect in Mandarin compounds due to the naming latencies for concrete compounds with two concrete constituents (cc condition) were much faster than concrete compounds with two abstract constituents (aa condition), which provided support for the decompositional model where constituents play important roles in the production of Mandarin compounds.

## Introduction

Research on the processing of morphologically complex words is well-established in the domain of language comprehension, where theoretical accounts are broadly divided into decompositional models (Koester et al., 2004, 2009; Longtin & Meunier, 2005; Rastle & Davis, 2008), full-listing models (e.g., Butterworth, 1983; Norris & McQueen, 2008), and hybrid models (Caramazza et al., 1988; Frauenfelder & Schreuder, 1991). Studies employing the morphological violation paradigm - for instance, presenting incorrect German past participles (e.g., *getanz-en instead of getanz-t) - have demonstrated distinct EEG responses to regular versus irregular verbs. This neurological dissociation supports hybrid models that posit different processing routes for regular and irregular morphology (Penke et al., 1997). Evidence for decomposition comes from studies on compound word processing where research on the time course of semantic integration in auditory compound comprehension has shown that constituents are integrated incrementally, aligning with a decompositional account (Koester et al., 2009).

A substantial body of work has focused on inflectional and derivational processes (Bozic & Marslen-Wilson, 2010; Marslen-Wilson et al., 1994; Penke et al., 1997; Rodriguez-Fornells et al., 2001; Smolka et al., 2015), with less attention given to the processing of compound words. This focus, however, diverges for Mandarin Chinese, where the study of compound representation has been a central concern from multiple theoretical and empirical perspectives, reflecting the dominant role of compounding in Mandarin morphology. A primary line of inquiry concerns the role of semantic transparency (Han et al., 2014; Liu & Peng, 1997; Peng et al., 1999; Su, 1998; Tsai, 1994). Researchers have also examined the influence of distributional properties, such as word frequency (Chen & Chen, 2006; Janssen et al., 2008; Peng et al., 1999; Taft, 1994; Yan et al., 2006; Zhang & Peng, 1992; Zhou & Marslen-Wilson, 1994) and transition probability between constituents (Myers & Gong, 2002; Taft, 1994). Additionally, studies have explored how compound-internal features - including morphological structure (Chung et al., 2010; Ji & Gagné, 2007; Liu, 2017) and headedness (Ceccagno & Basciano, 2007; Libben et al., 2003; Starosta et al., 1997) - affect processing.

In language production, however, two prominent models address the lexical representation of compound words during production processes: full-listing models (Butterworth, 1983; Caramazza, 1997; Dell, 1986) and decompositional models (Levelt et al., 1999), with hybrid models somewhat scarcer. According to the language production model proposed by Levelt et al. (1999), producing a word involves conceptual preparation, lexical access, phonological encoding, and articulation. Conceptual representations become activated when we engage in speech production, which then spreads to lexical representations linked to that concept. Before words can be articulated, phonological information is retrieved, involving word form encoding. According to full-listing models, compound words are stored holistically in the mental lexicon. In contrast, decompositional models suggest that compounds are represented and processed through their constituents. Both models are supported by empirical studies. For instance, to investigate the representation of compound words, Janssen et al. (2008) found that overall word frequency, rather than constituent frequency, influenced naming latencies in Mandarin compound words, supporting the full-listing hypothesis in language production. This was further supported by Bi et al. (2007), who showed that compound word frequency, not constituent morpheme frequency, affected the production performance of Chinese patients with aphasia. Additionally, Chen and Chen (2006) found that in Mandarin, naming latencies were not sensitive to constituent frequency, which aligned with the idea of a single lexical level between semantics and phonology.

In contrast, other studies support the decompositional model of compound word production. For instance, Roelofs (1996) and Bien et al. (2005) demonstrated that morpheme frequency and the morphological structure of compounds played a significant role in speech production planning, indicating that compound words are decomposed into their morphemes during production. Further evidence for the decompositional model comes from studies employing the long-lag priming paradigm (Kaczer et al., 2015; Koester & Schiller, 2008, 2011; Lensink et al., 2014; Verdonschot et al., 2012; Wang et al., 2024). This paradigm which was first demonstrated by Zwitserlood (2000, 2002), capitalizes on a key discovery: morphological priming effects persist across many intervening items, while semantic and phonological priming effects dissipate rapidly. In the critical priming trials, primes are selected to share either the first or second constituent morpheme with their corresponding targets (e.g., prime: sunshine; target: sunflower). According to a decompositional account of lexical representation, such morphological overlap should facilitate the processing of the target compound, because the shared constituent is pre-activated during prime processing. Therefore, the observation of a significant priming facilitation effect (e.g., faster reaction times) for morphologically related prime-target pairs, relative to unrelated control pairs, constitutes direct evidence in favor of morphological decomposition during compound word processing.

The research summarized above explores the representation of compound words in the mental lexicon of various languages in the process of production and comprehension. However, there are no consistent findings regarding this question. Examining the concreteness and abstractness of constituents to further explore their roles in compound production could offer valuable insights.

The concept of abstractness refers to ideas that are neither purely physically nor spatially constrained, making them generally more variable in their content across individuals and more challenging to associate with a single image than concrete concepts (Del Maschio et al., 2021). Compared to abstract words, concrete words are easier to comprehend, faster to read aloud and to evaluate for meaningfulness, and easier to remember (Belmore et al., 1982; Gerhand & Barry, 2000; Lee & Federmeier, 2008; Paivio, 1991; Schwanenflugel, 1991; Schwanenflugel et al., 1988). For instance, a concrete word like “table” is processed more quickly and accurately than an abstract word like “honesty” at the behavioral level. This preferential processing of concrete over abstract words is known as the concreteness effect (Holcomb et al., 1999; Kounios & Holcomb, 1994; West & Holcomb, 2000). Extending from single word to compounds processing, the concrete/abstract constituents could influence the retrieval of the compound words. For instance, consider the concrete compound words “church bell” and “apple core.” In “church bell,” both two constituents “church” and “bell” are concrete, whereas in “apple core,” the constituent “core” is relatively abstract. Therefore, the present study aimed to use the concreteness effect of constituents to investigate their influence on the retrieval of compound in Mandarin production.

There are studies examining concreteness effect in language production. For instance, research conducted by Hanley et al. (2013) used different paradigms to investigate the concreteness effect during language production. He designed two experiments to examine the impact of concreteness on word retrieval within sentence contexts. In Experiment 1, participants were asked to generate words from dictionary definitions. Results showed that abstract words were more challenging to retrieve, leading to more omissions and alternate responses than concrete words. Participants also experienced more tip-of-the-tongue (TOT) states when retrieving abstract words, indicating more phonological retrieval difficulties. In Experiment 2, participants generated words to complete sentences describing specific events. The number of abstract words recalled was significantly higher than that of concrete words in this task. The above literature showed that concreteness effects existed during language production and could serve as a robust method to investigate the research question in the present study.

Additionally, the neural mechanisms underlying the differences between concreteness and abstractness have been explored through the performance of patients with neural system damage, who often perform better on concrete than abstract words. For instance, in a study by Catricalà et al. (2014), patients with Alzheimer’s disease (AD) and the semantic variant of primary progressive aphasia (sv-PPA) completed tasks with controlled abstract and concrete stimuli. Results indicated that sv-PPA patients performed better with abstract than concrete concepts, showing category-specific effects: emotion concepts were preserved in AD, whereas social relations were selectively impaired in sv-PPA. Occasionally, AD patients displayed a living vs. non-living dissociation. These findings suggested that semantic memory disorders led to distinct patterns in abstract and concrete domains, highlighting differences in the brain regions affected by each condition.

Though much research has demonstrated that concrete expressions were processed more efficiently than abstract expressions across various languages and cognitive tasks, most of these studies have been conducted in language comprehension. Work has rarely been done in language production. One primary reason could be that it is hard in production tasks, for instance, picture naming, to present and name pictures of abstract concepts. Previous studies, for instance, Hanley et al. (2013) addressed this limitation using indirect methods, embedding concrete and abstract words within sentence contexts. The present study, however, aims to investigate the concreteness effect of constituents in Mandarin compound words during the production process by using two sets of concrete compounds as targets in a picture-naming task. At the same time, the concreteness of their constituents was manipulated to explore whether constituent concreteness affects Mandarin compound production while controlling for the overall concreteness of the compounds. Two conditions were created: the “aa” condition, where both constituents of the concrete compound were abstract, and the “cc” condition, where both constituents were concrete. It was predicted in the present study that naming latencies would be shorter for the “cc” condition compared to the “aa” condition due to the concreteness effect of the constituents at the behavioral level.

## Methodology

### Participants

Forty native right-handed Mandarin speakers, including six males, were recruited from Leiden University. The mean age was 24.05, with an SD of ± 2.31. All participants were from Mandarin-speaking provinces in China and spoke Mandarin as their mother tongue. Participants who had been living in the Netherlands for less than two years were included, while those who had been residing in the Netherlands for longer than two years were excluded from recruitment due to potentially higher proficiency in English and Dutch. All participants had normal or corrected-to-normal vision and received monetary compensation for their participation.

This study was approved by the Faculties of Humanities and Archaeology ethics committee at Leiden University (acceptance number: 2022/09). At the time of testing, none of the participants reported color blindness, learning disorders, hearing or visual impairments, or psychological or neurological conditions. Participants read an information sheet and provided informed consent by signing a consent form before the study began.

### Materials

In this study, we examined two conditions: the abstract (“aa”) condition, where the compound words were concrete, but their two constituents were abstract, and the concrete (“cc”) condition, where both the compound words and their constituents were concrete. Hence, only concreteness of the constituents varied between conditions. For example, in the “cc” condition, 河马 (/he2ma3/ “hippo”) is a concrete compound word with two concrete constituents: 河 (/he2/ “river”) and 马 (/ma3/ “horse”); in the “aa” condition, 乐队 (/yue4dui4/ “band”) is a concrete compound word with two abstract constituents: 乐 (/yue4/ “music”) and 队 (/dui4/ “team”). It is important to note that all target stimuli in both conditions were concrete disyllabic noun compounds. Forty-two Mandarin disyllabic compound nouns were selected as stimuli, with twenty-one words assigned to each condition.

We first calculated the concreteness and word frequencies of our stimuli by using two corpora to assess the concreteness and word frequency of the entire compound words in order to ensure no significant differences in concreteness and word frequency between two conditions.

For this, we utilized the MELD-SCH corpus, which provides concreteness and abstractness ratings for 9,877 two-character Mandarin Chinese words (Xu & Li, 2020), and SUBTLEX-CH corpus (Cai & Brysbaert, 2010), which provides word frequencies. We controlled for the concreteness of the compounds (*t* (40) < 1) based on MELD-SCH, as well as the compound frequency (*t* (40) < 1) using the SUBTLEX-CH corpus (see Table 1).

**Table 1:** The concreteness and word frequency calculation of compound words in our stimuli list

Corpus	Condition	Mean	
		Frequency (SD)	Concreteness (SD)
MELD-SCH & SUBTLEX-CH	aa	2.58 (0.48)	1.49 (0.18)
	cc	2.36 (0.46)	1.44 (0.14)
	*p-value*	0.14	0.30

To control for the concreteness and word frequency of constituents, we conducted a cross-corpus comparison based on two corpora. One was the Hong Kong Chinese Character Psycholinguistic Norms corpus (Su et al., 2023), which provides ratings for 4,376 individual Chinese characters on various factors, including word frequency, age of acquisition, familiarity, imageability, and concreteness. This corpus allowed for frequency and concreteness analysis for constituents.

The other corpus we used to calculate concreteness and frequency of constituents was from Liu et al. (2007), which provides word naming and psycholinguistic norms in Chinese, including information on familiarity, imageability, frequency (times/million), age of acquisition, and concreteness. By utilizing above corpora, we ensured a thorough control of the concreteness and word frequency of the constituents in our study.

We then ensured that each constituent’s concreteness was carefully controlled, confirming that the mean concreteness levels of constituents and the concreteness levels of each constituent differed significantly between the conditions. Furthermore, we controlled for each constituent’s frequency to ensure no significant differences across conditions (see Table 2). In addition, we controlled for other sub-lexical variables of the constituents, including stroke count (*t* (40) < 1) and imageability (*t* (40) < 1), ensuring that these factors did not significantly differ across the two conditions in the present study. Furthermore, the concreteness of the selected compounds and their constituents were rated by a group of 20 participants online, and their ratings (*t* (40) < 1) confirmed the validity of these chosen stimuli. See Appendix 1 for detailed stimuli list.

**Table 2:** The concreteness and word frequency calculation of constituents in our stimuli list

Corpus	Condition	1st Constituent		2nd Constituent		Mean for two constituents	
		Frequency(SD)	Concreteness (SD)	Frequency (SD)	Concreteness(SD)	Frequency (SD)	Concreteness (SD)
Su et al. (2023)	aa	2.54 (0.62)	4.10 (0.79)	2.25 (0.92)	4.30 (0.76)	2.39 (0.79)	4.20 (0.77)
	cc	2.47 (0.54)	6.44 (0.37)	1.97 (0.67)	6.06 (0.40)	2.22 (0.65)	6.24 (0.43)
	*p*	0.71	< 0.001	0.26	< 0.001	0.29	< 0.001
Liu et al. (2007)	aa	156.44 (424.83)	4.65 (0.62)	75.72 (123.56)	5.12(1.03)	116.08 (311.05)	4.88 (0.87)
	cc	187.21 (189.20)	6.62 (0.46)	57.79 (114.42)	6.57(0.34)	122.5 (167.58)	6.60 (0.40)
	*p*	0.78	< 0.001	0.65	< 0.001	0.91	< 0.001

We employed 42 colored pictures, with 21 pictures assigned to each condition in the present study. Thirty-four of these pictures were selected from the Multipic corpus (Duñabeitia et al., 2018), while eight were created by an illustrator in a similar style. The name agreement (*t* (40) < 1) and picture complexity (*t* (40) < 1) of these selected pictures were evaluated by another 20 participants online, following the method outlined by Snodgrass and Vanderwart (1980). In addition, we included ten filler pictures, which were used as warm-up trials at the beginning of each block, and as filler pictures during the experiment, with their frequencies and concreteness spanning a wide range. Participants were presented with 52 pictures in total, including 42 experimental pictures and 10 filler pictures.

### Design

The experiment in the present study employed a one-factorial within-subject design, with *Concreteness* as the fixed factor and *Subject* and *Item* as random factors. Participants engaged in a picture-naming task, where a pseudorandomized design was applied. Two versions of the pseudorandomized stimuli list were created during the experiment, with the first half of participants using the first version and the other half using the second version. Pictures from the same categories or those with the same phonological onset were not shown consecutively in the pseudorandomized design to avoid priming effects.

### Procedure

The experiment was presented using *E-prime 3.0* (Psychology Software Tools) and was conducted in a soundproof booth. Participants were seated in front of a computer in a dimly lit room. They used a microphone that was connected to a Chronos response device containing a voice key. The experiment consisted of three phases.

First, participants were given 10-15 min to familiarize themselves with target pictures and their names by studying a booklet at their own pace. Subsequently, the experimenter assessed whether participants correctly remembered the picture names by conducting a practice session through another booklet without their picture names. In this session, participants were asked to name all the target pictures as soon as possible, and the experimenter would correct them if they misnamed any pictures.

**Fig. 1:**
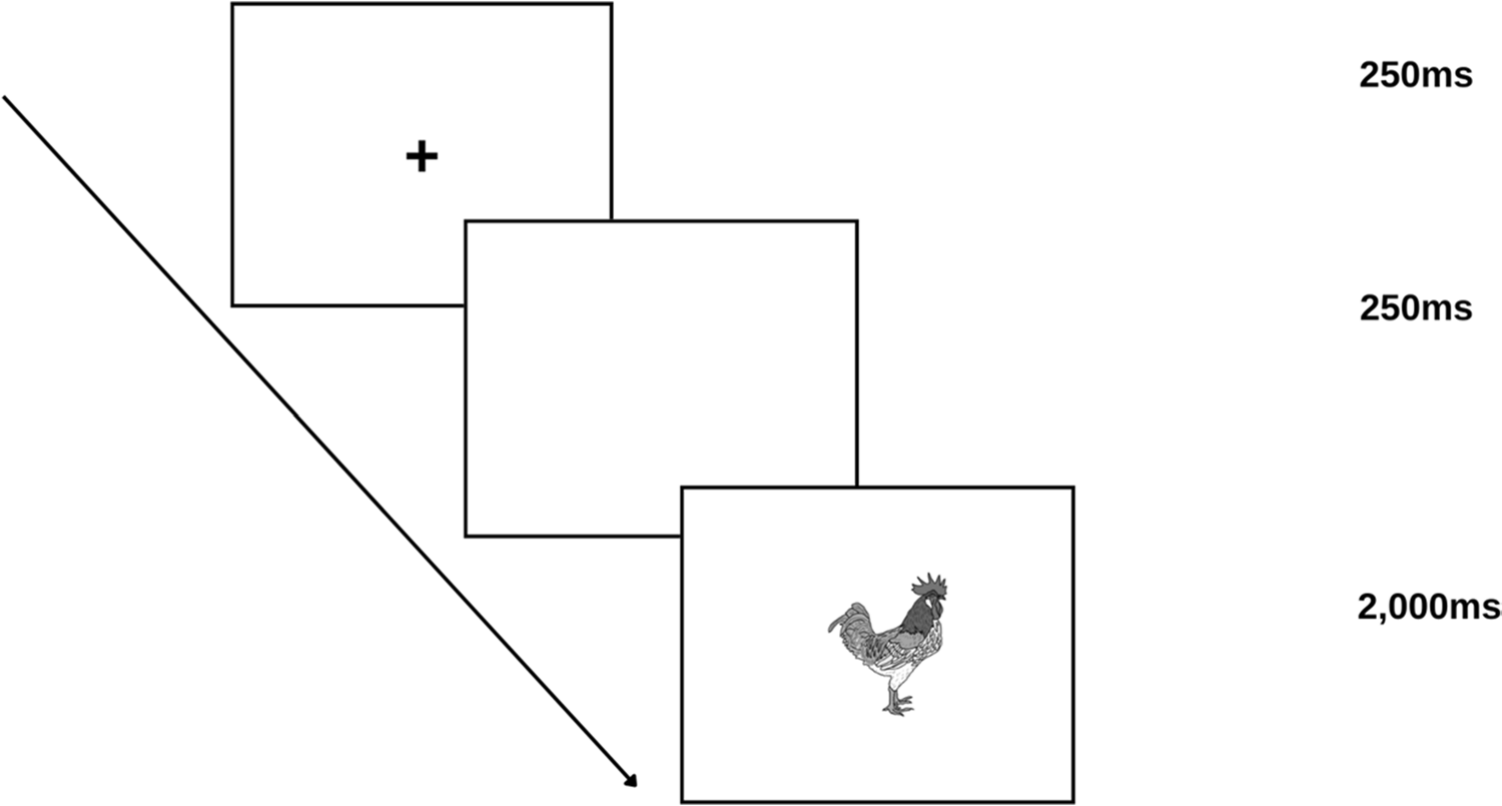
A trial sequence for the picture-naming task

In the experiment session, each trial began with a fixation cross displayed for 250 ms, followed by a blank screen for another 250 ms. Then, the target picture appeared in the center of the screen for 2,000 ms. Therefore, each trial lasted 2,500 ms, which was applied to all target and filler pictures. See Fig. 1 for a detailed demonstration of the experiment trial in the present study.

## Data analysis and results

### Data exclusion

For the analysis of reaction times (RTs), two types of trials were excluded: (1) error trials, in which an incorrect name was produced (1.49% of trials) by participants, and (2) outlier trials, defined as those with RTs exceeding 2.5 standard deviations above or below the participant’s mean RT for conditions (3.10% of remaining trials).

### Data analysis

**Fig. 2:**
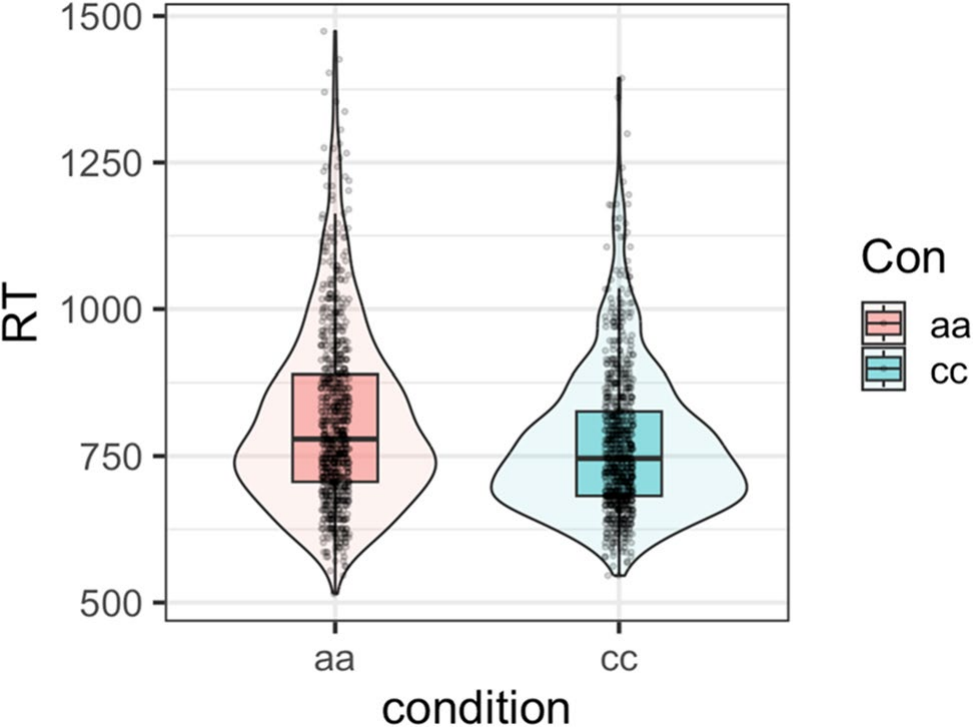
Mean naming latencies for each condition (*n* = 40)

Behavioral data were analyzed using *RStudio* Version 4.2.2. We first calculated descriptive statistics for naming latencies for each condition (see Table 3 and Fig. 2). Then, we employed a generalized linear mixed effect model (GLMM) (Lo & Andrews, 2015) using the *glmer ()* function from the *lme4* package with inverse Gaussian errors to model positively skewed RT data.

**Table 3:** Mean naming latencies (only correct trials were included) for each condition (*n* = 40)

Condition	Naming latencies (ms)	
	Mean	SD
aa	811.90	152.22
cc	770.63	123.91

To avoid over-parameterization and achieve a balance between Type-I error and statistical power, we employed a backward elimination strategy for the random effects structures of the model (Bates et al., 2015; Matuschek et al., 2017). Model comparisons and likelihood ratio tests were conducted at each step using the *anova()* function, evaluating Akaike’s Information Criterion (AIC) (Akaike, 1974), Bayesian Information Criterion (BIC) (Neath & Cavanaugh, 2012), and log-likelihood to determine whether the addition of a new factor significantly improved the model. 

In this analysis, *Concreteness* was included as a fixed effect, while *Subject* and *Item* were introduced as random effects. For naming latencies, the model of the best fit was *RT* ~ *Concreteness* + *(1* + *Concreteness | Subject)* + *(1 | Item)*. The results showed that the naming latencies of the “aa” and “cc” conditions significantly differed with *β* = -41.85 (95% CI [-57, -26.7]), *SE* = 7.73, *t* = -5.42, and *p* < 0.001. The by-subject random slope of concreteness in this model accounts for individual differences in the effect itself, showing that the effect of concreteness is not identical for all participants in the present study.

## Discussion and conclusion

The ongoing debate regarding the representation of compound words in our mental lexicon has led to numerous studies exploring this issue. The present study aimed to address this question by examining the concreteness effect of word constituents in compounds. Two conditions were established: “cc” condition representing concrete compound words with two concrete constituents and “aa” condition representing concrete compound words with two abstract constituents. The behavioral results in the present study showed significantly faster reaction times for the “cc” condition than the “aa” condition, suggesting concreteness effects of the constituents in Mandarin compound production.

The results reported in the present study have implications for the two models of lexical representation in production as discussed in *Introduction*. According to the full-listing model of lexical representations, compounds are stored in their full-listing format at the lexical level. In other words, the whole word is retrieved when naming the pictures instead of the individual constituents, which predicts no constituent effects. Contrastingly, the decompositional model predicts constituent effects due to the fact that each constituent is retrieved during compound production, which allows the possibilities of concrete constituent being retrieved faster than abstract constituents. Therefore, the concreteness effects of constituents found in the present study provide evidence for the decompositional hypothesis in the process of Mandarin compounds production.

Besides, the representation of multi-word units, including compounds, is a central topic in language processing research. While much of the evidence on this issue originates from studies of comprehension, the present investigation seeks to resolve this long-standing theoretical debate between decompositional (e.g., Levelt et al., 1999) and holistic (e.g., Caramazza, 1997) models of compound representation by shifting the empirical focus to language production, offering a critical, complementary testing ground for these competing theoretical claims. Testing the divergent hypotheses of compounds processing within the production domain is therefore theoretically imperative. A production task (e.g., picture naming task), by directly probing the stages of lexical access and phonological encoding, can reveal the underlying representational format - decomposed or holistic - free from potential parsing strategies that may dominate comprehension, which provides the primary rationale for investigating compound representation using a language production paradigm in the present study. Therefore, the resulting knowledge makes contributions to the existing literature in aspects of providing a direct test of model-specific production mechanisms.

Next, a critical, unresolved question is whether morphological decomposition is a central property of the lexicon or a task-specific strategy. If the production data align with patterns previously observed in comprehension (e.g., constituent frequency effects), this would support a domain-general linguistic principle that compounds are represented as morphologically structured units regardless of the processing direction (input vs. output). This would unify theories across comprehension and production. However, if the production data reveal a distinct pattern - for instance, showing whole-word dominance where comprehension studies normally show decomposition - it would instead argue for task-dependent or modality-specific representations. This outcome would necessitate more complex models where representational format is flexible or determined by processing constraints. Therefore, the discovery of a concreteness effect for morphological constituents in production in the present study, consistent with previous comprehension studies, supports domain-general morphological representation. By adjudicating between competing production models, testing the modality-generality of morphological effects, this study moves the field beyond descriptive debates toward a more integrated and mechanistic understanding of how complex words (especially compound words) are represented in our mental lexicon and planned for production.

The constituent-level concreteness effects we observed align with previous theoretical explanations including lexico-semantic weights hypothesis. This hypothesis claims that abstract words possess weaker semantic-lexical associations compared to concrete words as stated in previous studies (Hanley et al., 2004, 2013). For instance, Hanley et al. (2004) used Foygel and Dell’s (2000) speech production model to simulate imageability differences by varying lexico-semantic connection strengths. They posited that high-imageability words have stronger lexico-semantic association, while low-imageability words have weaker ones. In this model, lower association results in weaker activation of lexical units, causing more alternates and omissions for low-imageability words. Besides, in a definition task by Hanley et al. (2013), participants rated the most common alternatives to abstract target words as more compatible with definitions than those for concrete words, supporting this lexico-semantic weights hypothesis. In the present study, the facilitation of concrete constituents potentially show support for the above claim that it is easier for concrete concepts which have more distinctive semantic features and higher imageability to reach the activation threshold.

On the other hand, the concreteness effects have also been explained by alternative theoretical frameworks. For instance, Newton and Barry (1997) argued that abstract words are more difficult to retrieve in production because they face stronger competition from semantically related words. They proposed that retrieval issues for abstract words arise after activating the target word’s semantic representation. Since abstract words typically share many semantic features with other words, phonological representations of competing words are more likely to reach the activation threshold, increasing semantic errors. Although concrete words also activate competing items, their distinctive semantic features make them more likely to reach the activation threshold over competitors in tasks like word definition, reducing the likelihood of errors. Besides, Hoffman et al. (2011) found that abstract words are more ambiguous and have more senses than concrete words, as demonstrated through latent semantic analysis. The imageability effect observed in their study suggested that abstract words may be more difficult to process because they depend more on context for meaning, which is often lacking in experimental settings (Schwanenflugel et al., 1988).

Since the present results suggest that the explanatory frameworks offered for the single-word concreteness effect might provide a useful starting point or analytical lens for investigating the novel constituent-level effect we observed, these frameworks offer testable hypotheses for future research specifically designed to explore the mechanisms behind constituent concreteness in compounds production.

Additionally, since concreteness is a semantic feature, which is corresponding to the phase of conceptualization in the Levelt’s production model (1999), the present findings offer insights into models of lemma representation of compound words (Levelt et al., 1999; Marelli et al., 2012; Sprenger et al., 2006). According to Levelt et al.’s model (1999), compounds are initially represented as a single lemma, which is later decomposed at the lexeme level for phonological encoding. However, the concreteness effects observed in this study suggest that distinct lemma entries may exist for individual constituents during the production of Mandarin compound words. The fact that the concreteness of the constituents influences the production of compounds in the present study supports the hybrid lemma account proposed by Marelli et al. (2012) and Sprenger et al. (2006). The concreteness effects observed at the lemma level in the present study went against previous studies in Indo-European languages supporting a two-staged model (Mondini et al., 2004) with only a single lemma for word production. This difference could stem from cross-linguistic distinctions. First, Chinese writing is classified as a logographic system, where Chinese speakers may exhibit heightened lexical awareness attributed to the presence of Chinese characters and radicals, serving as visual cues. In addition, unlike alphabetic languages, Chinese characters do not directly correspond to phonemes but map onto meaningful morphemes in spoken language. This aspect implies that the regular or quasi-regular grapheme-phoneme conversions commonly found in alphabetic languages are not feasible in Mandarin Chinese (Plaut, 1996; Tan & Perfetti, 1998). Future studies should examine these points further.

It is also essential to acknowledge the limitations of this study. One such limitation is that it did not control for semantic transparency, a factor that has been more extensively investigated in the literature on comprehension, as noted earlier (Han et al., 2014; Liu & Peng, 1997; Peng et al., 1999; Su, 1998; Tsai, 1994). In language production, however, the distinction between opacity and transparency seems to have less impact in Indo-European languages based on previous literature (e.g., Koester & Schiller, 2008; but also see Zwitserlood, 2014). For instance, both opaque and transparent primes exhibit priming effects in compound production, as observed in the works of Koester and Schiller (2008). Nevertheless, there is a possibility that the obtained results may differ when applying to transparent or opaque words, suggesting that future research endeavors could also consider investigating opaque compound words in their research design.

Besides, we did not control for the Age of Acquisition (AoA) of compounds in the present study, but we conducted a post-hoc analysis to examine AoA as a potential confounding factor based on a corpus of Age of Acquisition ratings for 19,716 simplified Chinese words (Xu et al., 2021).^1^ The results revealed a significant influence of AoA for compounds themselves (*p* = 0.04) and for the mean AoA of both constituents (*p* = 0.0003). These findings indicate that AoA is likely to be a confounding variable in the observed concreteness effect, highlighting the need for future research to explicitly control for and investigate the nuanced role of AoA in this effect.

Furthermore, although we selected noun compounds as stimuli, we did not control for the word category (e.g., noun, verb, etc.) of their constituents. Hanley et al. (2013) highlighted the effects of word category on lexical retrieval in their two experiments. Therefore, future research should investigate this aspect further to provide more detailed evidence addressing the role of word class in compound words representation.

In conclusion, the present study’s results demonstrated that the concreteness of constituents probably plays a role in Mandarin compounds production and further supported the decompositional account instead of the full-listing account in Mandarin compounds production. The present study also highlights the importance of concreteness in language production along with some confounding factors, suggesting that future research could explore how concreteness influences language production more broadly.

## Data Availability

All data and analysis methods are available without restriction from the first author upon request.

## Appendix Experimental stimuli

**Table:**

Stimuli	Pinyin	English	Condition	English of each constituent
信封	xìnfēng	envelope	aa	信(letter) + 封(seal)
公主	gōngzhǔ	princess	aa	公(public) + 主 (master)
囚犯	qiúfàn	prisoner	aa	囚 (prisoner) + 犯 (criminal)
国王	guówáng	king	aa	国 (country) + 王 (king)
士兵	shìbīng	soldier	aa	士 (warrior) + 兵 (soldier)
彩虹	cǎihóng	rainbow	aa	彩(color) + 虹 (rainbow)
戒指	jièzhǐ	ring	aa	戒(warn) + 指 (finger)
抽屉	chōutì	drawer	aa	抽(pull) + 屉 (drawer)
拼图	pīntú	puzzle	aa	拼 (piece together) + 图 (picture)
教堂	jiàotáng	church	aa	教 (teach) + 堂 (hall)
日历	rìlì	calendar	aa	日(day) + 历 (calendar)
珍珠	zhēnzhū	pearl	aa	珍(precious) + 珠 (pearl)
皇冠	huángguàn	crown	aa	皇(royal) + 冠 (crown)
算盘	suànpán	abacus	aa	算(calculate) + 盘 (plate)
衬衫	chènshān	shirt	aa	衬(lining) + 衫 (shirt)
西装	xīzhuāng	suit	aa	西(west) + 装 (clothes)
贺卡	hèkǎ	greeting card	aa	贺 (congratulate) + 卡 (card)
邮差	yóuchāi	postman	aa	邮(mail) + 差 (messenger)
阳台	yángtái	balcony	aa	阳(sun) + 台 (platform)
音响	yīnxiǎng	audio	aa	音(sound) + 响 (loud)
领带	lǐngdài	tie	aa	领 (collar) + 带 (belt)
冰箱	bīngxiāng	refrigerator	cc	冰 (ice) + 箱 (box)
坟墓	fénmù	grave	cc	坟(grave) + 墓 (tomb)
天鹅	tiān'é	swan	cc	天 (sky/heaven) + 鹅 (goose)
手掌	shǒuzhǎng	palm	cc	手 (hand) + 掌 (palm)
树叶	shùyè	leaves	cc	树 (tree) + 叶 (leaf)
楼梯	lóutī	stairs	cc	楼(building) + 梯 (ladder)
水果	shuǐguǒ	fruit	cc	水 (water) + 果 (fruit)
河马	hémǎ	hippo	cc	河(river) + 马 (horse)
海鸥	hǎi'ōu	seagull	cc	海 (sea) + 鸥 (gull)
火箭	huǒjiàn	rocket	cc	火 (fire) + 箭 (arrow)
灯塔	dēngtǎ	lighthouse	cc	灯(lamp) + 塔 (tower)
牛奶	niúnǎi	milk	cc	牛 (cow) + 奶 (milk)
眼镜	yǎnjìng	glasses	cc	眼 (eye) + 镜 (lens)
花瓶	huāpíng	vase	cc	花(flower) + 瓶 (bottle)
药丸	yàowán	pill	cc	药(medicine) + 丸 (pill)
蛋糕	dàngāo	cake	cc	蛋 (egg) + 糕 (cake)
雨伞	yǔsǎn	umbrella	cc	雨(rain) + 伞 (umbrella)
雪山	xuěshān	snow mountain	cc	雪(snow) + 山 (mountain)
风车	fēngchē	windmill	cc	风(wind) + 车 (vehicle)
鸟笼	niǎolóng	birdcage	cc	鸟 (bird) + 笼 (cage)
龙虾	lóngxiā	lobster	cc	龙(dragon) + 虾 (shrimp)

## Footnotes

1 We would like to thank one of the reviewers for their suggestion of the analysis of Age of Acquisition (AoA).

## References

[CR1] Akaike, H. (1974). A new look at the statistical model identification. *IEEE Transactions on Automatic Control,**19*(6), 716–723. 10.1109/TAC.1974.1100705

[CR81] Bates, D., Kliegl, R., Vasishth, S., & Baayen, H. (2015). Parsimonious mixed models. arXivpreprint arXiv:1506.04967.

[CR84] Bates, D., Kliegl, R., Vasishth, S., & Baayen, H. (2015). Parsimonious mixed models. arXiv preprint arXiv:1506.04967.

[CR2] Belmore, S. M., Yates, J. M., Bellack, D. R., Jones, S. N., & Rosenquist, S. E. (1982). Drawing inferences from concrete and abstract sentences. *Journal of Verbal Learning and Verbal Behavior,**21*(3), 338–351. 10.1016/S0022-5371(82)90659-4

[CR4] Bien, H., Levelt, W. J., & Baayen, R. H. (2005). Frequency effects in compound production. *Proceedings of the National Academy of Sciences,**102*(49), 17876–17881. 10.1073/pnas.0508431102 10.1073/pnas.0508431102 PMC1287486 16301521

[CR3] Bi, Y., Han, Z., & Shu, H. (2007). Compound frequency effect in word production: Evidence from anomia. *Brain and Language,**103*(1–2), 55–56. 10.1016/j.bandl.2007.07.042

[CR5] Bozic, M., & Marslen-Wilson, W. (2010). Neurocognitive contexts for morphological complexity: Dissociating inflection and derivation. *Language and Linguistics Compass,**4*(11), 1063–1073. 10.1111/j.1749-818X.2010.00254.x

[CR6] Butterworth, B. (1983). Lexical Representation. In B. Butterworth (Ed.), *Language production: Vol. II. Development, writing and other language processes* (pp. 257–294). Academic Press.

[CR7] Cai, Q., & Brysbaert, M. (2010). SUBTLEX-CH: Chinese word and character frequencies based on film subtitles. *PLoS ONE,**5*(6), e10729. 10.1371/journal.pone.0010729 20532192 10.1371/journal.pone.0010729 PMC2880003

[CR8] Caramazza, A. (1997). How many levels of processing are there in lexical access? *Cognitive Neuropsychology,**14*(1), 177–208. 10.1080/026432997381664

[CR9] Caramazza, A., Laudanna, A., & Romani, C. (1988). Lexical access and inflectional morphology. *Cognition,**28*(3), 297–332. 10.1016/0010-0277(88)90017-0 3359755 10.1016/0010-0277(88)90017-0

[CR80] Catricalà, E., Della Rosa, P. A., Plebani, V., Vigliocco, G., & Cappa, S. F. (2014). Abstract and concrete categories? Evidences from neurodegenerative diseases. *Neuropsychologia*, *64*, 271–281. 10.1016/j.neuropsychologia.2014.09.041 25281886 10.1016/j.neuropsychologia.2014.09.041

[CR83] Catricalà, E., Della Rosa, P. A., Plebani, V., Vigliocco, G., & Cappa, S. F. (2014). Abstract and concrete categories? Evidences from neurodegenerative diseases. *Neuropsychologia*, *64*, 271–281. 10.1016/j.neuropsychologia.2014.09.041 25281886 10.1016/j.neuropsychologia.2014.09.041

[CR10] Ceccagno, A., & Basciano, B. (2007). Compound headedness in Chinese: An analysis of neologisms. *Morphology,**17*(2), 207–231.

[CR11] Chen, T.-M., & Chen, J.-Y. (2006). Morphological encoding in the production of compound words in Mandarin Chinese. *Journal of Memory and Language,**54*(4), 491–514. 10.1016/j.jml.2005.01.002

[CR12] Chung, K. K., Tong, X., Liu, P. D., McBride-Chang, C., & Meng, X. (2010). The processing of morphological structure information in Chinese coordinative compounds: An event-related potential study. *Brain Research,**1352*, 157–166. 20627093 10.1016/j.brainres.2010.06.069

[CR14] Dell, G. S. (1986). A spreading-activation theory of retrieval in sentence production. *Psychological Review,**93*(3), 283–321. 10.1037/0033-295X.93.3.283 3749399

[CR13] Del Maschio, N., Fedeli, D., Garofalo, G., & Buccino, G. (2021). Evidence for the concreteness of abstract language: A meta-analysis of neuroimaging studies. *Brain Sciences,**12*(1), 32. 10.3390/brainsci12010032 35053776 10.3390/brainsci12010032 PMC8773921

[CR15] Duñabeitia, J. A., Crepaldi, D., Meyer, A. S., New, B., Pliatsikas, C., Smolka, E., & Brysbaert, M. (2018). MultiPic: A standardized set of 750 drawings with norms for six European languages. *Quarterly Journal of Experimental Psychology,**71*(4), 808–816. 10.1080/17470218.2017.1310261 10.1080/17470218.2017.1310261 28326995

[CR82] Foygel, D., & Dell, G. S. (2000). Models of impaired lexical access in speech production. *Journal of Memory and Language*, *43*(2), 182–216. 10.1006/jmla.2000.2716

[CR85] Foygel, D., & Dell, G. S. (2000). Models of impaired lexical access in speech production. *Journal of Memory and Language*, *43*(2), 182–216. 10.1006/jmla.2000.2716

[CR16] Frauenfelder, U. H., & Schreuder, R. (1991). Constraining psycholinguistic models of morphological processing and representation: The role of productivity. In *Yearbook of Morphology* 1991 (pp. 165–183). Springer.

[CR17] Gerhand, S., & Barry, C. (2000). When does a deep dyslexic make a semantic error? The roles of age-of-acquisition, concreteness, and frequency. *Brain and Language,**74*(1), 26–47. 10.1006/brln.2000.2320 10924215 10.1006/brln.2000.2320

[CR19] Hanley, J. R., Dell, G. S., Kay, J., & Baron, R. (2004). Evidence for the involvement of a nonlexical route in the repetition of familiar words: A comparison of single and dual route models of auditory repetition. *Cognitive Neuropsychology,**21*(2–4), 147–158. 10.1080/02643290342000339 21038197 10.1080/02643290342000339

[CR20] Hanley, J. R., Hunt, R. P., Steed, D. A., & Jackman, S. (2013). Concreteness and word production. *Memory & Cognition,**41*, 365–377. 10.3758/s13421-012-0266-5 23104158 10.3758/s13421-012-0266-5

[CR18] Han, Y.-J., Huang, S.-C., Lee, C.-Y., Kuo, W.-J., & Cheng, S.-K. (2014). The modulation of semantic transparency on the recognition memory for two-character Chinese words. *Memory & Cognition,**42*(8), 1315–1324. 24894986 10.3758/s13421-014-0430-1

[CR21] Hoffman, P., Rogers, T. T., & Lambon Ralph, M. A. (2011). Semantic diversity accounts for the “missing” word frequency effect in stroke aphasia: Insights using a novel method to quantify contextual variability in meaning. *Journal of Cognitive Neuroscience,**23*, 2432–2446. 10.1162/jocn.2011.21614 21254804 10.1162/jocn.2011.21614

[CR22] Holcomb, P. J., Kounios, J., Anderson, J. E., & West, W. C. (1999). Dual-coding, context-availability, and concreteness effects in sentence comprehension: An electrophysiological investigation. *Journal of Experimental Psychology. Learning, Memory, and Cognition,**25*(3), 721-742. 10.1037/0278-7393.25.3.721 10368929 10.1037//0278-7393.25.3.721

[CR23] Janssen, N., Bi, Y., & Caramazza, A. (2008). A tale of two frequencies: Determining the speed of lexical access for Mandarin Chinese and English compounds. *Language and Cognitive Processes,**23*(7–8), 1191–1223. 10.1080/01690960802250900

[CR24] Ji, H., & Gagné, C. L. (2007). Lexical and relational influences on the processing of Chinese modifier-noun compounds. *The Mental Lexicon,**2*(3), 387–417.

[CR25] Kaczer, L., Timmer, K., Bavassi, L., & Schiller, N. O. (2015). Distinct morphological processing of recently learned compound words: An ERP study. *Brain Research,**1629*, 309–317. 10.1016/j.brainres.2015.10.029 26505918 10.1016/j.brainres.2015.10.029

[CR26] Koester, D., Gunter, T. C., Wagner, S., & Friederici, A. D. (2004). Morphosyntax, prosody, and linking elements: The auditory processing of German nominal compounds. *Journal of Cognitive Neuroscience,**16*(9), 1647–1668. 10.1162/0898929042568541 15601526 10.1162/0898929042568541

[CR27] Koester, D., Holle, H., & Gunter, T. C. (2009). Electrophysiological evidence for incremental lexical-semantic integration in auditory compound comprehension. *Neuropsychologia,**47*(8–9), 1854–1864. 10.1016/j.neuropsychologia.2009.02.027 19428417 10.1016/j.neuropsychologia.2009.02.027

[CR28] Koester, D., & Schiller, N. O. (2008). Morphological priming in overt language production: Electrophysiological evidence from Dutch. *NeuroImage,**42*(4), 1622–1630. 10.1016/j.neuroimage.2008.06.043 18674626 10.1016/j.neuroimage.2008.06.043

[CR29] Koester, D., & Schiller, N. O. (2011). The functional neuroanatomy of morphology in language production. *NeuroImage,**55*(2), 732–741. 10.1016/j.neuroimage.2010.11.044 21109010 10.1016/j.neuroimage.2010.11.044

[CR30] Kounios, J., & Holcomb, P. J. (1994). Concreteness effects in semantic processing: ERP evidence supporting dual-coding theory. *Journal of Experimental Psychology. Learning, Memory, and Cognition,**20*(4), 804–823. 10.1037/0278-7393.20.4.804 8064248 10.1037//0278-7393.20.4.804

[CR31] Lee, C.-L., & Federmeier, K. D. (2008). To watch, to see, and to differ: An event-related potential study of concreteness effects as a function of word class and lexical ambiguity. *Brain and Language,**104*(2), 145–158. 10.1016/j.bandl.2007.06.002 17659768 10.1016/j.bandl.2007.06.002 PMC2712631

[CR32] Lensink, S. E., Verdonschot, R. G., & Schiller, N. O. (2014). Morphological priming during language switching: An ERP study. *Frontiers in Human Neuroscience,**8*, 995. 10.3389/fnhum.2014.00995 25566022 10.3389/fnhum.2014.00995 PMC4264473

[CR33] Levelt, W. J., Roelofs, A., & Meyer, A. S. (1999). A theory of lexical access in speech production. *Behavioral and Brain Sciences,**22*(1), 1–38. 10.1017/s0140525x99001776 11301520 10.1017/s0140525x99001776

[CR34] Libben, G., Gibson, M., Yoon, Y. B., & Sandra, D. (2003). Compound fracture: The role of semantic transparency and morphological headedness. *Brain and Language,**84*(1), 50–64. 12537951 10.1016/s0093-934x(02)00520-5

[CR35] Liu, D. (2017). The influence of morphological structure information on the memorization of Chinese compound words. *Reading and Writing,**30*(8), 1813–1834.

[CR36] Liu, Y., & Peng, D.-L. (1997). Meaning access of Chinese compounds and its time course. *Cognitive processing of Chinese and related Asian languages* (pp. 219–232). The Chinese University Press.

[CR37] Liu, Y., Shu, H., & Li, P. (2007). Word naming and psycholinguistic norms: Chinese. *Behavior Research Methods,**39*(2), 192–198. 10.3758/BF03193147 17695344 10.3758/bf03193147

[CR39] Longtin, C.-M., & Meunier, F. (2005). Morphological decomposition in early visual word processing. *Journal of Memory and Language,**53*(1), 26–41. 10.1016/j.jml.2005.02.008

[CR38] Lo, S., & Andrews, S. (2015). To transform or not to transform: Using generalized linear mixed models to analyse reaction time data. *Frontiers in Psychology,**6*, 1171. 10.3389/fpsyg.2015.01171 26300841 10.3389/fpsyg.2015.01171 PMC4528092

[CR40] Marelli, M., Aggujaro, S., Molteni, F., & Luzzatti, C. (2012). The multiple-lemma representation of Italian compound nouns: A single case study of deep dyslexia. *Neuropsychologia,**50*(5), 852–861. 10.1016/j.neuropsychologia.2012.01.021 22300880 10.1016/j.neuropsychologia.2012.01.021

[CR41] Marslen-Wilson, W., Tyler, L. K., Waksler, R., & Older, L. (1994). Morphology and meaning in the English mental lexicon. *Psychological Review,**101*(1), 3–33. 10.1037/0033-295X.101.1.3

[CR42] Matuschek, H., Kliegl, R., Vasishth, S., Baayen, H., & Bates, D. (2017). Balancing type I error and power in linear mixed models. *Journal of Memory and Language,**94*, 305–315. 10.1016/j.jml.2017.01.001

[CR43] Mondini, S., Luzzatti, C., Zonca, G., Pistarini, C., & Semenza, C. (2004). The mental representation of verb-noun compounds in Italian: Evidence from a multiple single-case study in aphasia. *Brain and Language,**90*(1–3), 470–477. 10.1016/S0093-934X(03)00458-9 15172563 10.1016/S0093-934X(03)00458-9

[CR44] Myers, J., & Gong, S. (2002). Cross-morphemic predictability and the lexical access of compounds in Mandarin Chinese. *Folia Linguistica,**36*(1–2), 65–96.

[CR45] Neath, A. A., & Cavanaugh, J. E. (2012). The Bayesian information criterion: Background, derivation, and applications. *Wires Computational Statistics,**4*(2), 199–203. 10.1002/wics.199

[CR46] Newton, P. K., & Barry, C. (1997). Concreteness effects in word production but not word comprehension in deep dyslexia. *Cognitive Neuropsychology,**14*, 481–509. 10.1080/026432997381457

[CR47] Norris, D., & McQueen, J. M. (2008). Shortlist B: A Bayesian model of continuous speech recognition. *Psychological Review,**115*(2), 357–395. 10.1037/0033-295X.115.2.357 18426294 10.1037/0033-295X.115.2.357

[CR48] Paivio, A. (1991). Dual coding theory: Retrospect and current status. *Canadian Journal of Psychology = Revue Canadienne De Psychologie,**45*(3), 255–287. 10.1037/h0084295

[CR49] Peng, D., Liu, Y., & Wang, C. (1999). How is access representation organized? The relation of polymorphemic words and their morphemes in Chinese. In J. Wang, A. W. Inhoff, & H.-C. Chen (Eds.), *Reading Chinese script: A cognitive analysis* (pp. 65–89). Lawrence Erlbaum Associates Publishers.

[CR50] Penke, M., Weyerts, H., Gross, M., Zander, E., Münte, T. F., & Clahsen, H. (1997). How the brain processes complex words: An event-related potential study of German verb inflections. *Cognitive Brain Research,**6*(1), 37–52. 10.1016/S0926-6410(97)00012-8 9395848 10.1016/s0926-6410(97)00012-8

[CR51] Plaut, D. C. (1996). Relearning after damage in connectionist networks: Toward a theory of rehabilitation. *Brain and Language,**52*(1), 25–82. 10.1006/brln.1996.0004 8741976 10.1006/brln.1996.0004

[CR52] Rastle, K., & Davis, M. H. (2008). Morphological decomposition based on the analysis of orthography. *Language and Cognitive Processes,**23*(7–8), 942–971. 10.1080/01690960802069730

[CR53] Rodriguez-Fornells, A., Clahsen, H., Lleó, C., Zaake, W., & Münte, T. F. (2001). Event-related brain responses to morphological violations in Catalan. *Cognitive Brain Research,**11*(1), 47–58. 10.1016/S0926-6410(00)00063-X 11240111 10.1016/s0926-6410(00)00063-x

[CR54] Roelofs, A. (1996). Morpheme frequency in speech production: Testing WEAVER. *Yearbook of morphology 1996* (pp. 135–154). Kluwer.

[CR55] Schwanenflugel, P. J. (1991). Contextual constraint and lexical processing. In G. B. Simpson (Ed.), Understanding word and sentence (pp. 23–45). North-Holland. 10.1016/S0166-4115(08)61528-9

[CR56] Schwanenflugel, P. J., Harnishfeger, K. K., & Stowe, R. W. (1988). Context availability and lexical decisions for abstract and concrete words. *Journal of Memory and Language,**27*(5), 499–520. 10.1016/0749-596X(88)90022-8

[CR57] Smolka, E., Gondan, M., & Rösler, F. (2015). Take a stand on understanding: Electrophysiological evidence for stem access in German complex verbs. *Frontiers in Human Neuroscience,**9*, Article 62. 10.3389/fnhum.2015.00062 25767442 10.3389/fnhum.2015.00062 PMC4341544

[CR58] Snodgrass, J. G., & Vanderwart, M. (1980). A standardized set of 260 pictures: Norms for name agreement, image agreement, familiarity, and visual complexity. *Journal of Experimental Psychology: Human Learning and Memory,**6*(2), 174–215. 10.1037/0278-7393.6.2.174 7373248 10.1037//0278-7393.6.2.174

[CR59] Sprenger, S. A., Levelt, W. J., & Kempen, G. (2006). Lexical access during the production of idiomatic phrases. *Journal of Memory and Language,**54*(2), 161–184. 10.1016/j.jml.2005.11.001

[CR60] Starosta, S., Kuiper, K., Ng, S. S., & Wu, Z.-Q. (1997). On Chinese compounding and Chinese VR compounds. In *New approaches to Chinese word formation: Morphology, phonology and the lexicon in modern and ancient Chinese* (Vol. 105, pp. 347–376). Berlin, New York: Mouton de Gruyter.

[CR62] Su, I.-F., Yum, Y. N., & Lau, D.K.-Y. (2023). Hong Kong Chinese character psycholinguistic norms: Ratings of 4376 single Chinese characters on semantic radical transparency, age-of-acquisition, familiarity, imageability, and concreteness. *Behavior Research Methods,**55*(6), 2989–3008. 10.3758/s13428-022-01928-y 36002627 10.3758/s13428-022-01928-y PMC10558066

[CR61] Su, Y.-C. (1998). *The representation of compounds and phrases in the mental lexicon: Evidence from Chinese*. *University of Maryland Working Papers in Linguistics*, *6,*179–199.

[CR63] Taft, M. (1994). The influence of character frequency on word recognition responses in Chinese. In H.-W. Chang, J.-T. Huang, C.-W. Hue, & O. J. L. Tzeng (Eds.), *Advances in the study of Chinese language processing* (Vol. 1, pp. 59–73). National Taiwan University.

[CR64] Tan, L. H., Liu, H.-L., Perfetti, C. A., Spinks, J. A., Fox, P. T., & Gao, J.-H. (2001). The neural system underlying Chinese logograph reading. *NeuroImage,**13*(5), 836–846. 11304080 10.1006/nimg.2001.0749

[CR65] Tan, L.-H., & Perfetti, C. A. (1998). Phonological codes as early sources of constraint in Chinese word identification: A review of current discoveries and theoretical accounts. *Reading and Writing,**10*, 165–200.

[CR66] Tsai, C.-H. (1994). *Effects of semantic transparency on the recognition of Chinese two-character words: Evidence for a dual-process model* [Master’s thesis, National Chung Cheng University, Chia-Yi, Taiwan].

[CR67] Verdonschot, R. G., Middelburg, R., Lensink, S. E., & Schiller, N. O. (2012). Morphological priming survives a language switch. *Cognition,**124*(3), 343–349. 10.3758/BF03197127 22743054 10.1016/j.cognition.2012.05.019

[CR68] Von Grebmerzu Wolfsthurn, S., Robles, L. P., & Schiller, N. O. (2021a). Cross-linguistic interference in late language learners: An ERP study. *Brain and Language,**221*, 104993. 10.1016/j.bandl.2021.104993 34303111 10.1016/j.bandl.2021.104993

[CR69] Von Grebmerzu Wolfsthurn, S., Robles, L. P., & Schiller, N. O. (2021b). Noun-phrase production as a window to language selection: An ERP study. *Neuropsychologia,**162*, 108055. 10.1016/j.neuropsychologia.2021.108055 34626618 10.1016/j.neuropsychologia.2021.108055

[CR70] Wang, J., Schiller, N. O., & Verdonschot, R. G. (2024). Morphological encoding in language production: Electrophysiological evidence from Mandarin Chinese compound words. *PLoS ONE,**19*(10), e0310816. 10.1371/journal.pone.0310816 39356709 10.1371/journal.pone.0310816 PMC11446431

[CR71] West, W. C., & Holcomb, P. J. (2000). Imaginal, semantic, and surface-level processing of concrete and abstract words: An electrophysiological investigation. *Journal of Cognitive Neuroscience,**12*(6), 1024–1037. 10.1162/08989290051137558 11177422 10.1162/08989290051137558

[CR72] Xu, X., & Li, J. (2020). Concreteness/abstractness ratings for two-character Chinese words in MELD-SCH. *PLoS ONE,**15*(6), e0232133. 10.1371/journal.pone.0232133 32569306 10.1371/journal.pone.0232133 PMC7307783

[CR73] Xu, X., Li, J., & Guo, S. (2021). Age of acquisition ratings for 19,716 simplified Chinese words. *Behavior Research Methods,**53*, 558–573. 10.3758/s13428-020-01455-8 32748240 10.3758/s13428-020-01455-8

[CR74] Yan, G., Tian, H., Bai, X., & Rayner, K. (2006). The effect of word and character frequency on the eye movements of Chinese readers. *British Journal of Psychology,**97*(2), 259–268. 16613652 10.1348/000712605X70066

[CR75] Zhang, B. & Peng, D. (1992). Decomposed storage in the Chinese lexicon. In *Advances in psychology* (Vol. 90, pp. 131–149).

[CR76] Zhou, X., & Marslen-Wilson, W. (1994). Words, morphemes and syllables in the Chinese mental lexicon. *Language and Cognitive Processes,**9*(3), 393–422.

[CR77] Zwitserlood, P. (2014). The role of semantic transparency in the processing and representation of Dutch compounds. In *Morphological Structure, Lexical Representation and Lexical Access (RLE Linguistics C: Applied Linguistics)* (pp. 341–368). Routledge. 10.4324/9781315857213

[CR78] Zwitserlood, P., Bölte, J., & Dohmes, P. (2000). Morphological effects on speech production: Evidence from picture naming. *Language and Cognitive Processes,**15*(4–5), 563–591.

[CR79] Zwitserlood, P., Bölte, J., & Dohmes, P. (2002). Where and how morphologically complex words interplay with naming pictures. *Brain and Language,**81*(1–3), 358–367. 12081405 10.1006/brln.2001.2530

